# Rehospitalisation rates after long-term follow-up of patients with severe mental illness admitted for more than one year: a systematic review

**DOI:** 10.1186/s12888-023-05290-x

**Published:** 2023-10-27

**Authors:** Sayaka Sato, Miharu Nakanishi, Makoto Ogawa, Makiko Abe, Naonori Yasuma, Toshiaki Kono, Momoka Igarashi, Mai Iwanaga, Takayuki Kawaguchi, Sosei Yamaguchi

**Affiliations:** 1 Department of Community Mental Health & Law, National Center of Neurology and Psychiatry (NCNP), National Institute of Mental Health, 4-1-1 Ogawa-Higashi, Kodaira, Tokyo 187-8553 Japan; 2 Department of Psychiatric Nursing, Tohoku University Graduate School of Medicine, Miyagi, Japan

**Keywords:** Psychiatric services, Systematic reviews, Schizophrenia, Administration, Community mental health

## Abstract

**Aims:**

This study aimed to conduct a systematic review of studies on the outcomes of long-term hospitalisation of individuals with severe mental illness, considering readmission rates as the primary outcome.

**Methods:**

Studies considered were those in which participants were aged between 18 and 64 years with severe mental illness; exposure to psychiatric hospitals or wards was long-term (more than one year); primary outcomes were readmission rates; secondary outcomes were duration of readmission, employment, schooling, and social participation; and the study design was either observational or interventional with a randomised controlled trial (RCT) design. Relevant studies were searched using MEDLINE, PsycINFO, Web of Science, CINAHL, and the Japan Medical Abstract Society. The final search was conducted on 1 February 2022. The risk of bias in non-randomised studies of interventions was used to assess the methodological quality. A descriptive literature review is also conducted.

**Results:**

Of the 11,999 studies initially searched, three cohort studies (2,293 participants) met the eligibility criteria. The risk of bias in these studies was rated as critical or serious. The 1–10 years readmission rate for patients with schizophrenia who had been hospitalised for more than one year ranged from 33 to 55%. The average of readmission durations described in the two studies was 70.5 ± 95.6 days per year (in the case of a 7.5-year follow-up) and 306 ± 399 days (in the case of a 3–8-year follow-up). None of the studies reported other outcomes defined in this study.

**Conclusions:**

The readmission rates in the included studies varied. Differences in the follow-up period or the intensity of community services may have contributed to this variability. In countries preparing to implement de-institutionalisation, highly individualised community support should be designed to avoid relocation to residential services under supervision. The length of stay for readmissions was shorter than that for index admissions. The results also imply that discharge to the community contributes to improved clinical outcomes such as improved social functioning. The validity of retaining patients admitted because of the risk of rehospitalisation was considered low. Future research directions have also been discussed.

**Supplementary Information:**

The online version contains supplementary material available at 10.1186/s12888-023-05290-x.

## Introduction

Deinstitutionalisation, transitioning psychiatric patients from institutional settings to community-based care, remains a contemporary and pressing issue. According to the World Mental Health Report, there is increasing emphasis on promoting psychiatric care within the community and reducing prolonged hospital stays [27]. Globally, the number of psychiatric patients hospitalised for over a year in psychiatric wards has declined [26]. However, in regions such as the Americas and Eastern Mediterranean countries, more than 25% of all inpatients with psychiatric disorders have stays exceeding one year [26]. Long-term hospitalisation and its challenges in transitioning to community-based care have surfaced as national concerns in countries such as India, Singapore, China, and South Korea since the 2010s [2, 3, 15, 20, 28].

Historically, by the 1970s, Western countries such as the United States and parts of Europe had primarily achieved de-institutionalisation, showing optimistic outcomes for reintegrated patients [6, 21]. Systematic reviews in the 2000s further indicated that patients' social functions, psychiatric symptoms, and quality of life were not only maintained but also improved post-discharge, with notably low rates of homelessness, incarceration, and suicide [11, 23]. However, despite these positive observations, hesitation persists among healthcare professionals regarding the viability of community reintegration. Although numerous studies have detailed long-term hospitalized patients' clinical characteristics and outcomes, there is little literature concerning readmission rates.

The assessment of the readmission rate is vital because it serves as an accessible and reliable index for psychiatric care [17]. The readmission rate index may provide insights beyond mere rehospitalisation, encompassing aspects such as patients' psychiatric symptoms, skills, and quality of life post-discharge [19]. Moreover, recent epidemiological studies have focused on large-scale data analysis of readmission rates, underscoring their significance for clinical and policy considerations [5, 9, 12]. However, all these studies analysed big data at the national or state level and considered short-term hospital admissions. Research on long-term psychiatric inpatients is limited.

Given the continued emphasis on de-institutionalisation in certain parts of the world, it is essential to understand the readmission rates of long-term hospitalised patients who will likely be targeted for support during de-institutionalisation. Such insights are critical for regions actively transitioning or planning to transition patients from long-term institutional care to community-based settings.

Therefore, we conducted a systematic review focusing on the readmission rates of psychiatric patients with a history of long-term admission (> 1 year), including those from non-Western regions. We aimed to provide up-to-date evidence to healthcare professionals and policymakers to improve clinical practice and develop well-informed policies.

## Methods

This study was based on the updated reporting guidelines for systematic reviews (Preferred Reporting Items for Systematic Reviews and Meta-Analysis [PRISMA] 2020 statement) [16]. The systematic review protocol was registered in the UMIN-CTR (registration number: UMIN000040254).

### Inclusion and exclusion

The inclusion and exclusion criteria were based on study design, participants, exposure, and outcomes. The language was restricted to English and Japanese, and no restrictions were imposed on the publication year. Observational and randomised controlled trial (RCT)-based interventional studies were included in the study design. Only data from the control group (the group that received the usual treatment) were included in the RCT. Studies involving individuals aged 18–64 years diagnosed with severe mental illness (schizophrenia, bipolar disorder, or major depression) at baseline were included. Exposure was defined as admission to a psychiatric hospital for at least one year. Studies were excluded if they had only individuals with mood disorders, anxiety, dementia, addiction, eating habits, personality, intellectual disabilities, or developmental disorders. In contrast, we included patients whose diagnoses were comorbid with severe mental illness, as described above. Studies that combined these diagnoses with severe mental illness were included only if data on severe mental illness could be extracted. Furthermore, studies in which participants had received specific community services beyond the usual support (e.g. intensive case management and home-visiting services) were excluded because they could not be considered natural outcomes. The primary outcome was the readmission rate, and the secondary outcomes were readmission duration, employment status, schooling, and social participation.

### Search and selection procedure

We searched for relevant studies using MEDLINE, PsycINFO, Web of Science, CINAHL, and the Japan Medical Abstracts Society (containing Japanese articles). Two authors and an experienced librarian reviewed the keywords and terms used in the thesaurus search and developed a search formula. (See Appendix for details). We conducted the initial searches of each database in October 2019 and February 2022, and the final search was conducted in June 2023. In addition, manual searches were conducted on the references of eligible studies determined through a database search and screening. At least two authors independently conducted database searches.

Similarly, at least two reviewers individually screened the study records from the databases using titles and abstracts. We obtained full-text articles from the study records identified as potentially relevant by one of them. Two reviewers individually assessed the eligibility of each full-text article. In addition, cited reference searches were conducted on the references of eligible studies. During this process, whenever a conflict in their views arose, the two reviewers discussed and agreed on a joint conclusion or sought the opinion of a third researcher (SY) when the disagreement could not be resolved.

### Assessment of methodological quality

The methodological quality of the studies included in this review was examined using the Risk of Bias in Nonrandomised Studies of Interventions [13], which assessed seven domains:1) confounding factors, 2) selection of study participants, 3) classification of exposure, 4) deviation from intended direction, 5) missing data, 6) measurement of outcomes, and 7) selection of reported outcomes on a scale of Low, Moderate, Serious, and Critical. At least two reviewers individually rated the risk of bias for each study and determined the final score through discussion.

### Outcome, data extraction, and data synthesis

Following the inclusion criteria, we extracted information on the readmission rate, readmission length, employment status, schooling, and social participation as outcomes. If the necessary information could not be found in the articles, the first author (SS) asked the corresponding author of each article for this information. Given the inclusion criteria of this review (hospitalised in a psychiatric ward for at least one year), we expected to incorporate studies from older patients and observe heterogeneity in the description of outcome variables. Therefore, we determined that a meta-analysis was not feasible and conducted a descriptive review. The first author (SS) extracted the outcome-related descriptions from each eligible study and compiled them into tables, whereas the other authors (MIg and MIw) independently reviewed the results.

## Results

### Selection process

**Fig. 1:**
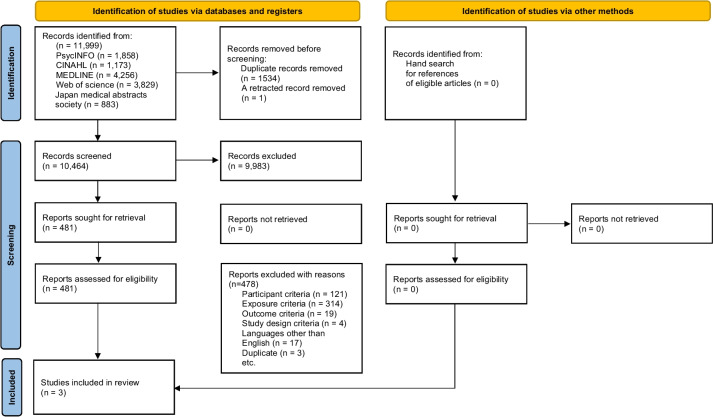
PRISMA flow diagram

In total, 11,999 articles were extracted from each database. After excluding duplicate and retracted records, 10,464 articles were screened based on the title and abstract. After initial screening, we obtained 481 full-text articles and reassessed their eligibility. Three eligible studies were included in the systematic review (Fig. 1).

### Characteristics and methodological quality of the included studies

All the studies employed cohort designs [8, 14, 24]. The total number of participants in these studies was 2293, and approximately 63% (*n* = 1446) were male. The proportion of people with schizophrenia was 100% in two studies [8, 24]. Among the participants in Okin et al.’s study, 70% were diagnosed with schizophrenia [14]. The mean or median age of the patients was 30 s and 40 s. Winkler et al. [24] did not clearly describe the mean or median age of participants. Therefore, we calculated the approximate median value using the class value and number of persons provided in their article. The studies were conducted in the USA, Japan, and the Czech Republic. Two studies were conducted in the 1980s [8, 14] and one from the 1990s to the 2010s [24]. The maximum average stay at the index admission was 11.5 years [14]; the duration of follow-up ranged from 1 to 10 years, and the follow-up rates ranged from 74 to 100%. Okin et al. [14] identified variables other than primary and secondary outcomes, including psychiatric symptoms, social functioning, and duration from discharge to hospital readmission. Information was obtained through peer assessments by support workers and references to medical records. Regarding assessing the risk of bias, two out of three studies were rated 'Critical', and one was rated 'Serious'. Confounding bias may have affected the results. The ROBIN I-based risk-of-bias ratings are listed in Table 1.

**Table 1:**
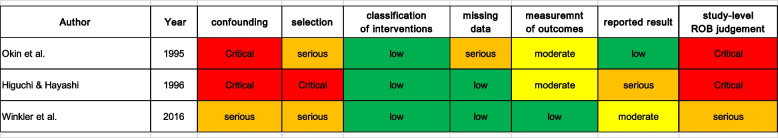
Risk of bias assessed using ROBINS-I

### Outcomes

#### Readmission

The readmission rates for each eligible study were 32.5% one year after discharge [24], 51.2% at 3–8 years after discharge [8], and 54.7% at 4–10 years after discharge [14]. Regarding readmission duration, two studies described the averages of readmission duration: Okin et al. [14] reported an average length of rehospitalisation of 70.5 ± 95.6 days per year over a mean follow-up period of 7.5 years; Higuchi and Hayashi [8] found that the average rehospitalisation was 306 ± 399 days during the 3–8-year follow-up period. In the latter study, the exact average number of readmission days was unclear because the follow-up period differed for each participant. Therefore, we used the median follow-up period (5.5 years) in our calculations and estimated the average number of readmission days per year to be less than 60 days. Winkler et al. [24] did not describe the readmission duration.

### Employment status, schooling, and social participation

None of the eligible studies investigated the association between long-term hospitalisation and employment, schooling, or social participation. Higuchi and Hayashi [8] showed the employment rate immediately after discharge from index admission. However, we did not discuss this because it is unlikely to represent the outcomes of long-term hospitalisation. A summary of the outcomes is presented in Table 2.

**Table 2:**
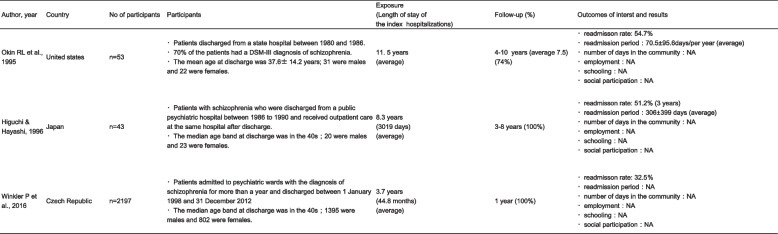
Description of the included studies [8, 14, 23, 24]

## Discussion

This review reports the outcomes of patients with schizophrenia-related illnesses who were hospitalised for more than one year, based on three studies from the USA, the Czech Republic, and Japan. One country has completed de-institutionalisation (the United States), and the other two still have many psychiatric beds (the Czech Republic and Japan). While the evidence level of the three studies was not high, this review found that the readmission rates of those studies were approximately 33–55% when long-term patients with schizophrenia-related illnesses were followed up for 1–10 years. The average readmission length for patients discharged to the community was estimated to be 60–70 days per year in two studies conducted in the USA and Japan. Employment, schooling, and social participation were not mentioned in any studies included in this review.

### Evidence level

The level of evidence for the studies included in this review was low. In particular, a high risk of bias (study-level ROB judgement, ‘critical’) was reported in three studies published until the 2000s. Research methodologies and reporting guidelines were developed and disseminated over the past decade. In other words, the publication year of each study may have affected methodological and reporting quality. In addition, the low methodological quality of the individual studies suggests caution when interpreting the results.

The rehospitalisation rates across the three studies varied from approximately 33% to 55%,with this variation likely stemming from differing follow-up periods. Comparing these readmission rates to those in earlier studies presented challenges. For example, both Okin et al. [14] and Higuchi and Hayashi [8] reported readmission rates exceeding 50%. Nonetheless, Okin et al. [14] had a 7.5-year follow-up period, whereas Higuchi and Hayashi's follow-up period was 5.5 years [8]. This suggests that individuals in the former study could remain in the community without being hospitalised for a more extended period, possibly due to the community-based services they received. The participants in Okin et al.’s [14] study were discharged to residential services with supervisors, whereas those in Higuchi and Hayashi's [8] Japanese study lived alone or with their families. In addition, they perhaps did not receive sufficient community care because the focus on community care in Japan began only in the twenty-first century [10]. Similarly, Winkler et al. [24] reported a readmission rate of > 30% with a year follow-up in the Czech Republic. Although they did not report service utilisation data, the Czech community mental health care system was inadequate, and the length of hospitalisation was excessively long [25]. In summary, both the follow-up period and quality of community care appeared to affect the readmission rate between studies.

Compared to studies not included in this review, long-term follow-up American and British cohort studies conducted in the 1970s-80 s showed that 67% of people with schizophrenia were readmitted at least once during the 5-year follow-up period and 79% during the 14-year follow-up period [1, 18]. These studies recruited patients admitted for less than one year and had a shorter average stay for index admission than the studies included in this review. In addition, 14% and 21% of the patients in the two studies were admitted several times [1, 18], suggesting that there were patients with revolving doors. Indeed, de-institutionalisation was underway, although community care was indigent in the 1970s–80 s in the United States. However, the readmission rates reported by Okin et al. [14], a study included in this review, are low. Relevant research suggests that the readmission rates of psychiatric patients are associated with the continuity of care from inpatient treatment to community care and the quality of community services [5, 22]. These differences in readmission rates based on when the studies were conducted may be due to the level of community services in the year each study was conducted.

Two of the included studies presented data on the length of rehospitalisation [8, 14]. Okin et al. [14] showed an average of 70.5 ± 95.6 readmission days per year after discharge from the index hospitalisation; Higuchi and Hayashi [8] found an average readmission of 306 ± 399 days in the three years after discharge from the index hospitalisation. Since the number of follow-up days differed for each participant in both studies, Okin et al.’s study was standardised. Simultaneously, Higuchi and Hayashi’s study was the weighted average obtained by dividing the total number of readmission days for all participants admitted three years after discharge by the number of participants. This is substantially fewer days considering the average length of stay in the United States (6.3 ± 7.5 years in 1991, only hospitalisations of more than one year) and Japan (606.1 days in 1996, without limitation on index admission of the length of stay) during the years in which the studies were conducted. [4, 7]. A potential reason for this difference may be selection bias. For example, Okin et al. [14] and Higuchi and Hayashi [8], which were included in this review, might have been conducted in areas that had more adequate services after discharge, compared to the studies by Desai et al. [4] and the Ministry of Health and Welfare [7]. Another assumption is that the sample characteristics may vary significantly between studies. Although it is impossible to accurately estimate a single factor, the length of rehospitalisation can vary widely between areas and hospitals, even during the same period.

None of the studies included in this review addressed employment status, education, or social participation. Okin et al. [14] and Higuchi and Hayashi [8] published studies in the 1990s before social and personal recovery in psychiatry became widespread. Therefore, the outcome measures were mainly clinical conditions and functions measured using scales, and hard outcomes related to social participation were not collected. Winkler et al. [24] also analysed nationwide medical data but did not mention variables not collected beforehand. Future prospective cohort studies with sophisticated designs that include a collection of variables related to personal recovery in line with the current values are warranted.

### Strengths and limitations

This study is the first systematic review of outcomes for patients with severe mental illness who have had an admission history of more than a year for more than one year, focusing on readmission rates, a hard outcome, as the primary outcome. This review also included studies conducted in Western and non-Western countries. As several Eastern European and Asian countries still have many psychiatric beds, the results of this review can help encourage mental health care reforms in these countries by projecting the number of beds needed for readmitted patients and designing community care services. However, this study had some limitations. Another limitation was the small number of included studies. Furthermore, significant heterogeneity was observed among these studies in terms of the duration of the index admission, post-discharge follow-up period, and methodologies used for calculating the follow-up period. Such discrepancies impeded our ability to draw consistent and robust conclusions. While the review endeavoured to incorporate evidence from various regions, it was confined to studies published in English and Japanese. Combining studies with shorter index hospitalisations, brief follow-up periods, and those published in languages other than English may have expanded the pool of articles, thereby allowing for more comprehensive and conclusive insights.

## Conclusion

This systematic review investigated readmission outcomes in patients with severe mental illness who were hospitalised for more than one year. The 1- to 10-year readmission rates were 33–55% among the three studies. One possible reason for high readmission rates may be the availability of community services. In countries that address de-institutionalisation, it is desirable to design systems that provide highly individualised community support beyond relocating to residential services with a supervisor. The length of stay for readmissions was shorter than that for index admissions, suggesting that discharge from the hospital to the community contributes to improvements in clinical outcomes other than psychiatric symptoms, such as improved social functioning reported by themselves. However, the limited number of studies and the substantial heterogeneity pose challenges in deriving conclusive results. Refining the inclusion criteria and conducting systematic reviews encompassing a greater volume of research papers is advisable. Moreover cohort studies should integrate outcomes pertinent to individual recovery, including employment and educational attainment, using more robust and rigorous methodologies.

### Supplementary Information

**Additional file 1.** Search formulae.

## Data Availability

Data supporting the findings of this study are available from the corresponding author [SS] upon reasonable request.
