# Supplementary material for: Rehospitalisation rates after long-term follow-up of patients with severe mental illness admitted for more than one year: a systematic review
Source: BMC Psychiatry. 2023 Oct 27;23:788. doi: 10.1186/s12888-023-05290-x (PMC10612306; doi:10.1186/s12888-023-05290-x)
Supplement: Supplementary file 1 — Additional file 1. Search formulae. [file 12888_2023_5290_MOESM1_ESM.docx]

**Supplementary**

**Medline with full text via EBSCO**

1 MH ‘Mental Disorders+’

2 MH ‘Hospitalisation+’

3 MH ‘Patient Readmission’

4 MH ‘patient admission+’

5 MH ‘Employment+’

6 MH ‘Work+’

7 MH ‘Social Participation’

8 OR/3-7

9 MH ‘Prospective Studies’

10 MH ‘Cohort Studies+’

11 MH ‘Observational Studies as Topic’

12 PT ‘Observational Study’

13 intervention

14 MH ‘trial’

15 random*

16 control group

17 comparison

18 comparative

19 OR/9-18

20 1 AND 2 AND 8 AND 19

**PsycINFO**

1 exp mental disorders/

2 hospital*.af.

3 exp psychiatric patients/

4 2 OR 3

5 exp treatment duration/

6 Occupations

7 Community Involvement

8 OR/5-7

9 ‘Longitudinal Studies’[th]

11 exp followup studies/

12 exp intervention/

13 exp clinical trials/

14 ‘random*’.af.

15 ‘control group’.af.

16 comparison.af.

17 comparative.af.

18 OR/9-17

19 1 AND 4 AND 8 AND 18

**Web of science**

1 schizophr*

2 psychiat*

3 psychos*

4 bipolar

5 depressi*

6 severe mental illness

7 mental

8 OR/1-7

9 admission

10 admitted

11 hospitali*

12 discharge

13 hospital stay

14 OR/9-13

15 hospital*

16 ward*

17 15 AND 16

18 psychiatric

19 14 AND 17 AND 18

20 readmission

21 readmitted

22 ‘length of stay’

23 ‘community stay’

24 ‘hospital stay’

25 employ*

26 job

27 work

28 ‘social participation’

29 OR/20-28

30 prospective

31 longitudinal

32 cohort

33 observational

34 follow-up

35 national wide

36 database

37 OR/30-36

38 8 AND 19 AND 29 AND 37

**CINAHL**

1 ‘Mental Disorders’[TH]

2 Hospitalisation[TH]

3 Deinstitutionalisation[TH]

4 ‘Patient Discharge’[TH]

5 OR/2-4

6 Readmission[TH]

7 ‘Patient Admission’[TH]

8 Employment[TH]

9 Work[TH]

10 ‘Social Participation’[TH]

11 ‘return to work’

12 ‘Length of Stay’[TH]

13 OR/6-12

14 ‘Longitudinal Study’

15 ‘Nonexperimental Studies’[TH]

16 ‘Experimental Studies’[TH].

17 random*

18 ‘control group’

19 comparison

20 comparative

21 OR/14-20

22 1 AND 5 AND 13 AND 21

Note: The Japan Medical Abstracts Society is a Japanese language search database that does not list the search formulae. Please send an e-mail to the corresponding author if you want to obtain more details.
